# Chemotherapy-induced bowel ischemia: diagnostic imaging overview

**DOI:** 10.1007/s00261-021-03024-9

**Published:** 2021-04-03

**Authors:** Alfonso Reginelli, Angelo Sangiovanni, Giovanna Vacca, Maria Paola Belfiore, Maria Pignatiello, Giuseppe Viscardi, Alfredo Clemente, Fabrizio Urraro, Salvatore Cappabianca

**Affiliations:** Department of Precision Medicine, University of Campania “L. Vanvitelli,”, 80138 Naples, Italy

**Keywords:** Bowel toxicity, Computed tomography, Chemotherapy, Adverse drug reactions

## Abstract

Cancer patients need multimodal therapies to treat their disease increasingly. In particular, drug treatment, as chemotherapy, immunotherapy, or various associations between them are commonly used to increase efficacy. However, the use of drugs predisposes a percentage of patients to develop toxicity in multiple organs and systems. Principle chemotherapy drugs mechanism of action is cell replication inhibition, rapidly proliferating cells especially. Immunotherapy is another tumor therapy strategy based on antitumor immunity activation trough agents as CTLA4 inhibitors (ipilimumab) or PD-1/PD-L1 inhibitors as nivolumab. If, on the one hand, all these agents inhibit tumor growth, on the other, they can cause various degrees toxicity in several organs, due to their specific mechanism of action. Particularly interesting are bowel toxicity, which can be clinically heterogeneous (pain, nausea, diarrhea, enterocolitis, pneumocolitis), up to severe consequences, such as ischemia, a rare occurrence. However, this event can occur both in vessels that supply intestine and in submucosa microvessels. We report drug-related intestinal vascular damage main characteristics, showing the radiological aspect of these alterations. Interpretation of imaging in oncologic patients has become progressively more complicated in the context of “target therapy” and thanks to the increasing number and types of therapies provided. Radiologists should know this variety of antiangiogenic treatments and immunotherapy regimens first because they can determine atypical features of tumor response and then also because of their eventual bowel toxicity.

## Introduction

Cancer patients need multimodal therapies to treat their disease increasingly. In particular, drug treatment, as chemotherapy, immunotherapy, or various associations between them are commonly used to increase efficacy. However, the use of drugs predisposes a percentage of patients to develop toxicity in multiple organs and systems [1]. They can show themselves as mild or with severe symptoms, often not recognizable by clinical examination alone. Antitumor drug therapy is nowadays based on the use of different agents with specific actions; we can distinguish several categories mainly: standard chemotherapy, molecularly targeted therapies, immunotherapy. Principle chemotherapy drugs mechanism of action is cell replication inhibition, rapidly proliferating cells especially. They usually interfere with DNA and RNA synthesis through different tools depending by the drug used: cisplatin has a DNA intercalating action, fluorouracil is an antimetabolite, vincristine a mitotic spindle inhibitor, cyclophosphamide is an alkylating agent, gemcitabine an antimetabolite [2]. Sometimes, targeted therapies induced cell apoptosis through modifying intracellular proteins or tumor growth pathways inhibition such as neoangiogenesis [3]. Immunotherapy is another tumor therapy strategy based on antitumor immunity activation trough agents as CTLA4 inhibitors (ipilimumab) or PD-1/PD-L1 inhibitors as nivolumab [4]. If, on the one hand, all these agents inhibit tumor growth, on the other, they can cause various degrees toxicity in several organs, due to their specific mechanism of action. A representative scheme was reported by Viswanathan et al. [3]. Particularly interesting are bowel toxicity, which can be clinically heterogeneous (pain, nausea, diarrhea, enterocolitis, pneumocolitis), up to severe consequences, such as ischemia, a rare occurrence. However, this event can occur both in vessels that supply intestine and in submucosa microvessels. This thesis is supported by proof that even disorders such as enteritis or perforation recognize a vascular origin [5]; as reported in two series, bevacizumab-induced perforation is caused by interference with bowel microvasculature, resulting in onset vessels ischemia and thrombosis in 0.9–4.4% of cases [6, 7]. We report drug-related intestinal vascular damage main characteristics, showing the radiological aspect of these alterations.

## Intestinal toxicity: which anticancer drugs cause it?

Common side effects during chemotherapy are observed in gastrointestinal system and consists of mucosal damage mainly, which can manifest itself with various symptoms [8]. At the cellular level, toxicity is due to apoptosis mostly induced by involved drug-specific mechanisms of small intestine crypts cells [9, 10]. Although nausea and vomiting are the most frequent gastrointestinal symptoms for many chemotherapy regimens, their onset is not directly related to the intestinal mucosa damage, but to stimulation of serotonergic receptors of the chemoreceptor trigger zone in the fourth cerebral ventricle [11, 12]. Diarrhea is another common chemotherapy toxic effect; the pathophysiology of chemotherapy-induced diarrhea is not well understood and is likely multifactorial [1]. Chemotherapic agents most involved in diarrhea onset are 5-fluorouracil and irinotecan [13, 14]. SN-38, an active metabolite of irinotecan, induces direct mucosal damage as a mechanism of delayed diarrhea [15]. The mitotic phase blocking of intestinal crypt cells, as well as villous destruction and reabsorption surface reduction, can be involved in 5-FU induced diarrhea [16]. Other drugs, as vinca alkaloids, are responsible for bowel dilatation frequently; its pathophysiology can be caused by autonomic nervous system induced-neurotoxicity [17]. Likewise, docetaxel can lead to bowel damage; sporadic but severe ischemic colitis docetaxel-related cases are reported in the literature [18, 19]. Many molecular target drugs are responsible for intestinal toxicity, some of the critical severity: first bevacizumab, related to bowel pneumatosis, bleeding, and perforation [20]. Pneumatosis can also be caused by cisplatin and irinotecan, and it referred to transmural ischemia, in the presence of portomesenteric venous gas mostly [21, 22]. This condition is uncommon, but it is related to mortality up to 75% of cases [23]. Anti-EGFR agents, as erlotinib or cetuximab, frequently cause enteritis for direct damage to the intestinal epithelium [24]. The most common side effect of anti-EGFR and anti-VEGF agents is diarrhea, which appears in up to 40% of patients who underwent to these treatment [25, 26]. Immune checkpoint inhibitors are related to a series of side effects termed immune-related adverse events (irAEs). Immunotherapy-related enterocolitis has features similar to graft-versus-host disease [27], presenting in different patterns (diffuse colitis, segmental colitis associated with diverticulitis and isolated recto-sigmoid colitis) related to the appearance of diarrhea (44%), colitis (18%), bowel perforation (< 1%) [28, 29]. Although these drugs have a good tolerability profile, cases of bowel perforations caused by BRAF and MEK inhibitors/anti-CTLA-4 and anti-PD-1 antibody association are reported in the literature [30].

## Indirect bowel damage: vessel toxicity drugs-related

Many anticancer drugs can induce vascular toxicity, venous, and more rarely arterial system. Great and microvessels may be affected by thrombosis and thromboembolism; if bowel vessels are involved, they can give rise to critical ischemic events, which lead to necrosis and perforation. Thrombotic and thromboembolism events pathogenesis is induced by drug-related endothelial damage, with consequent basement membrane exposure and abnormal coagulation cascade activation [31, 32]. Platinum-based chemotherapy regimens are responsible for higher thromboembolism risk, but not other vascular events; this risk is superimposable between cisplatin and carboplatin [33]. Although gemcitabine prothrombotic mechanisms are still mostly unknown, it is responsible for increased thrombotic risk both in small peripheral vessels and in large draining veins of parenchymal organs [34]. Coagulation cascade hyperactivation as also been observed in several case series where thrombotic risk increases in patients undergoing cisplatin/gemcitabine association have been evaluated [35, 36]. Many molecular target agents can increase thrombotic risk: thalidomide, an immunomodulatory and antiangiogenetic drug commonly used in multiple myeloma therapy, has shown thromboembolic events raise [36, 37]; anti-VEGFR and anti-VEGF targeted agents as bevacizumab, sorafenib and sunitinib are responsible to arterial thrombotic events increase, because of their role in endothelial integrity regulation probably [31]. Their correlation with arterial thromboembolic events is shown in several case series, where mesenteric artery thromboembolism is described [38–40]. Thromboembolisms and vasculitis caused by immune checkpoint regulators such as anti-PDL1 seem to be rare events [41]; however, in described cases in the literature, their onset is related to very severe presentations [42–44].

## Imaging modalities and features

Clinical features of acute ischemic bowel is very changeable: acute abdominal pain could be determined by many different causes (pancreatitis, coeliac disease, duodenal ulcers, irritable bowel syndrome) and the ‘classic triad’ of chronic mesenteric ischemia (postprandial pain, weight loss and an abdominal bruit) is rarely found in clinical practice [45].

Radiological imaging is crucial in the emergency setting. It is very difficult to make differential diagnosis only through physical examination; as explained by Terlouw et al., at least upper gastrointestinal endoscopy and abdominal imaging (computed tomography/magnetic resonance imaging) must be executed if there is a suspicious of chronic mesenteric ischemia. Colonoscopy should be performed in patients with diarrhea.

In the emergency setting the baseline of radiological examination in patient with acute abdominal pain include abdominal ultrasonography (US) and/or abdominal plain radiography. US allows to detect some differential diagnosis of acute abdominal pain such as cholecystitis, pancreatitis, hernias. Colour and Power Doppler imaging therefore could be useful to study bowel’s vascularity: an increase of vascularity of the bowel wall and adjacent mesentery is a sign of hyperemia and inflammatory bowel disease. On the contrary, a reduction of vascularity is a specific sign of ischemia [46]. Duplex ultrasound might be used for screening to evaluate an eventual significant proximal mesenteric artery stenosis: in this case, an additional CTA or MRA imaging is mandatory [45]. Plain radiography might present some warning signs of ischaemic colitis disease such as “thumbprinting”, which appears as rounded opacities near the sides of a gas-filled distended colon, loss of haustration and dilation of the colonic lumen; obviously is also possible to discover signs of advanced pathology such as intramural gas “pneumatosis linearis”, portal venous gas, megacolon and pneumoperitoneum [47].

According to Mazzei et al., MRI could play an important role not only for follow up but also for the diagnosis of acute ischemic colitis without using contrast medium through combined T2-weighted steady state free procession sequences on coronal plane and T2W fast-recovery fast-spin echo sequences both in coronal and axial plane. For example, colonic wall thickening is typical both on CT and MR in case of severe colonic: especially when this sign has segmental distribution is quite specific for ischemic colitis compared to inflammatory colitis [48]. MRI is useful especially in patients with impaired renal function or in patient with previous allergic reactions to iodine contrast agents avoiding radiation exposure. However, compared to CT, MRI shows some difficulties when performed in the emergency setting because of its long time of execution even if the spreading of newer performing scanners could overcome this problem in future.

Currently, computed tomography (CT) represents the primary imaging technique in imaging bowel injuries with numerous advantages in comparison with other diagnostic modalities [49]. In particular it may diagnose bowel injuries especially in the emergency setting, thanks to its panoramic evaluation of abdominal organs, vessels, and intestinal lumen and wall; CT can also determine the eventual presence of inflammatory collections [50].

## CT findings

Mainly, multidetector CT represents the gold standard for the intestinal study because, in few minutes, it can provide a complete study of the abdomen also in non-cooperating patients and can avoid angiography through a multiphase study using contrast agents.

First, we know that oncologic patients are almost exclusively examined by CT to detect their “tumor burden” so it is possible to discover intestinal pathological features in asymptomatic patients during the planned follow-up; it is known that 70.8% of cancer patients with pneumatosis and/or bowel perforation are asymptomatic [49]. If symptomatic, it is essential to keep in mind that the principal symptom of bowel pathology and ischemia is just represented by abdominal pain, followed by diarrhoea and vomit. These symptoms are incredibly nonspecific, and they could be related to predicted effects that can appear near the administration of chemotherapy drugs and regimens. Gastrointestinal pathological processes (diverticulitis, appendicitis, enteritis, colitis, intestinal occlusions) should be recognized as soon as possible in the emergency setting because they may lead to bowel ischemia, necrosis, and perforation; dissimilar chronic mesenteric ischemia, surgery remains standard treatment of acute mesenteric ischemia. CT features of bowel ischemia and necrosis are almost similar in spite of the primary cause [50]. First is necessary to establish the correct etiology among acute arterial mesenteric ischemia (AAMI), acute venous mesenteric ischemia (AVMI), non-occlusive mesenteric ischemia (NOMI), ischemia/reperfusion injury (I/R), ischemic colitis (ischemic and reperfusive form) [51–56]. Mesenteric arterial occlusion or mesenteric venous thrombosis occlusion configure an arterial or venous ischemic injury, respectively. In the acute arterial mesenteric setting, the damaged small bowel loops are contracted in consequence of spastic reflex ileus, and the intestinal wall presents absence/weak enhancement. At an advanced phase, bowel wall thins, show a “paper-thin” aspect, [50, 51]; intestinal loops appear only gas-filled and dilated (hypotonic ileus), and peritoneal free fluid may be discovered. Air–fluid levels occur when hypotonic reflex ileus evolves into paralytic ileus [52]. The most specific feature of bowel ischemia would seem to be the absence or deficient enhancement of the bowel wall, a CT finding today more easily demonstrable using the dual energy technique (DECT) [56]. Likewise, intramural gas (intestinal pneumatosis) is a particular CT feature, but it is uncommon; intramural gas is due to luminal gas penetration into the bowel wall through the damaged mucosa [54, 55]. Another unusual CT feature of bowel ischemia is mesenteric or portal venous gas and depicts the extension of intramural gas into the mesenteric venous system. However, both of these CT signs, when present, are indicative of an advanced phase of ischemia so they should be considered as makers of severity.

**Fig. 1:**
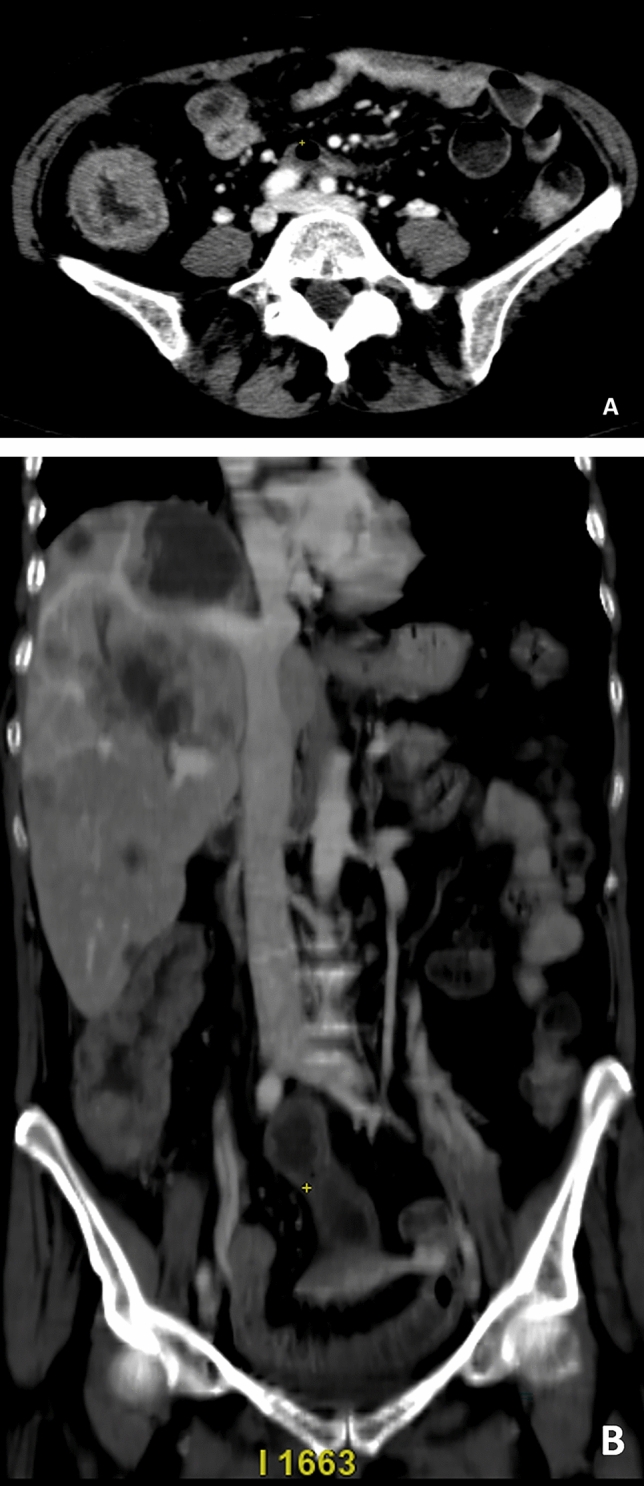
Axial and coronal CT imaging show diffuse wall thickening bowel with or without evidence of the “target sign” and heightened enhancement of the thickened bowel wall: this event is common but nonspecific for bowel ischemia (**a** and **b**)

Free intraperitoneal gas means perforation of an infarcted bowel segment [57]. In the venous ischemic setting (AVMI) is possible to discover focal or diffuse wall thickening bowel with or without evidence of the “target sign” and heightened enhancement of the thickened bowel wall: this event is common but nonspecific for bowel ischemia (Fig. 1). Venous congestion caused by blood stasis can be highlighted as mesenteric veins engorgement, and the mesenteric fat may show increased values of attenuation because of mesenteric edema [56]. On unenhanced CT scans, presence of hypo-density of bowel loops wall is evocative of intramural edema; meanwhile, hyper-density could reveal mesenteric veins engorgement, congestion and hemorrhage of bowel loops wall. NOMI includes the causes of mesenteric ischemia without a sign of occlusion of the mesenteric artery or vein in the region of bowel necrosis [58–62]. In the small intestine, CT and surgical findings might be similar to AAMI ones only in hyperacute settings; NOMI could be followed indeed by a reperfusion phenomena that tries to overcome the ischaemic injury caused by hypoperfusion [58]. In this case, bowel wall thickening is determined by the presence of oedema, vascular congestion and granulation tissue that are typical features of any acute inflammatory process. On unenhanced CT phase is possible to detect bowel wall thickening and its high attenuation probably due to the intramural haemorrhage and haemorrhagic infarction resulting from vascular congestion [63]. Reperfusion event determines also a regional increase of mesenteric fat density (misty mesentery) with or without the presence of mesenteric fluid [58]. DECT might help the differential diagnosis between ischemic and nonischemic intestinal segments using iodine maps and 40-keV monoenergetic images, respectively [56]; particularly, it is useful for the detection of reperfusion setting. Reduction in bowel wall attenuation on DECT is almost indicative for arterial occlusive ischemia (AAMI or NOMI) without reperfusion phenomena; on the contrary, bowel wall involved in AVMI, AAMI and NOMI followed by reperfusion shows hyper-attenuation [56]. In addition, DECT offers many advantages; its iodine sensitivity allows to reduce contrast medium dose and concentration avoiding the risk of acute kidney injury in patients with chronic kidney disease or in old patients with associated comorbidities [56].

Radiologists must take in count that bowel ischemia can also exist in malignant conditions, such as in proximity to a colonic carcinoma or a metastatic intestinal lesion [53].

Moreover, some chemotherapy agents may determine spontaneous gastrointestinal necrosis and even perforation [63, 64]. At CT examination, chemotherapy-induced enteropathy consists mainly of focal or diffuse bowel wall thickening or as regional mesenteric vascular congestion [53]; sometimes bowel perforation appears.

Side effects from molecular target therapy include enterocolitis, pneumatosis, micro-perforations of the bowel, fistula formation, and wound dehiscence, especially in rectal carcinoma [57, 65, 66].

These features, however, are not specific because they can also occur after radiation that induces small-vessel occlusions and could determine ischemia anywhere in the digestive tract, especially in patients with prior abdominal surgery with adhesive changes, previous peritonitis before radiation therapy and in patients that have cardiovascular factors or risk [67]. Immunotherapy use is spreading rapidly and radiologists should consider the possibility of side effects caused by these medications. Patel et al. reported a case of a metastatic melanoma 56-year-old man undergoing immunotherapy treatment first with ipilimumab and after with nivolumab that was affected by small bowel perforation secondary to nivolumab and ipilimumab related tumor regression [68]. CT scan of the abdomen and pelvis showed a perforation of the jejunum and consequent pneumoperitoneum. The perforation site was localized near to the metastatic lesion that was reduced for dimensions thanks to the rapid response to immunotherapy. According to Patel and his group, the patient’s response to nivolumab and ipilimumab was so drastic that the tumor shrinkage caused by the medications determine perforation within the small bowel [68].

**Fig. 2:**
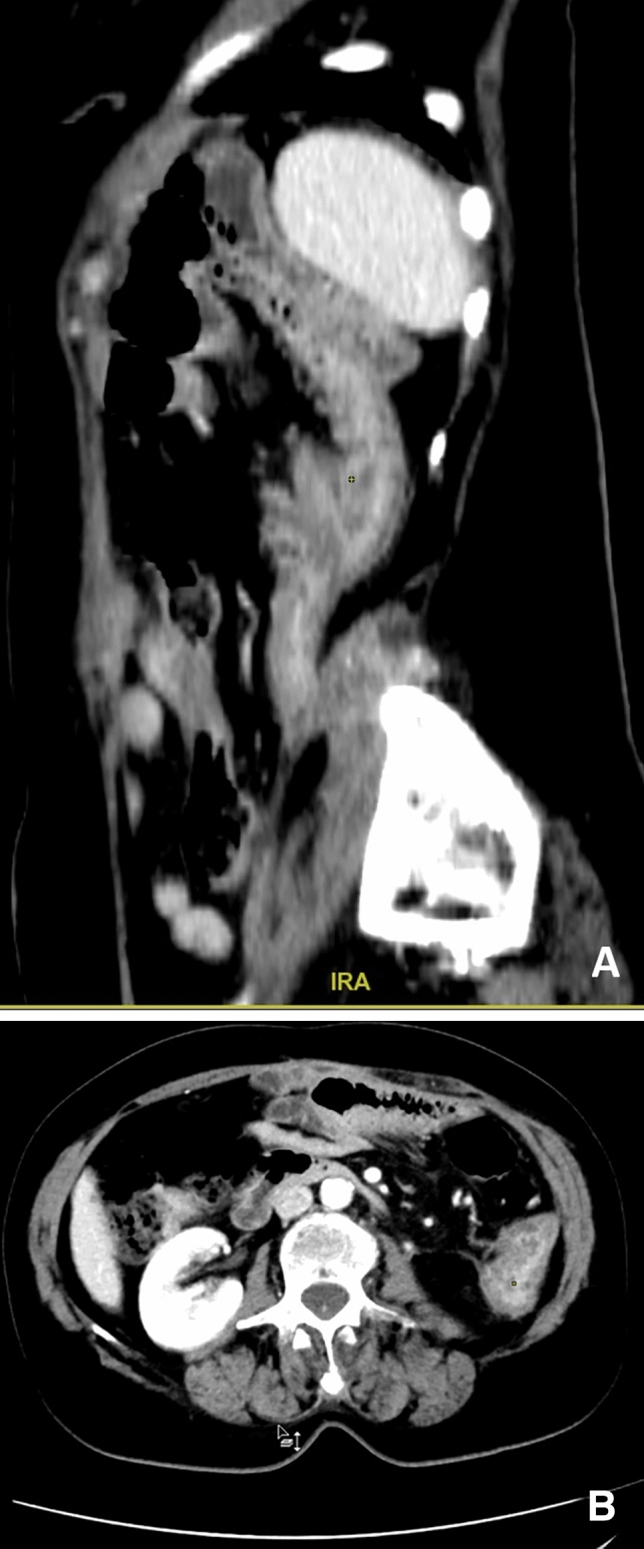
CT imaging in coronal and axial plane (**a** and **b**). Abdominal CT with IV iodinated contrast showed sigmoid diverticulitis with colonic wall thickening and pericolic fat stranding

We report the case of a 35 years old male patient diagnosed in February 2017 with squamous non-small-cell lung cancer (sqNSCLC). From June 2017, he received a second-line treatment with nivolumab for advanced disease achieving a major partial response. After 23 drug administrations, he presented with symptoms of moderate left lower quadrant pain without fever. Abdominal CT with IV iodinated contrast showed sigmoid diverticulitis with colonic wall thickening and pericolic fat stranding (Fig. 2). The patient was managed conservatively with low dose steroids and antibiotic treatment (ceftriaxone and metronidazole) [69]. After full recovery of symptoms, the patient resumed an immune checkpoint inhibitor four weeks later.

## Discussion

Cancer patients have an increased thromboembolic risk involving up to 50% of their total; many events during disease evolution remain unknown, and their diagnosis occurs at the autopsy [70, 71]. Chemotherapy is responsible for thromboembolic risk increase, such as showed in an epidemiologic study [71]; as described above, the pathophysiology of this increased risk is multifactorial [72, 73]. Where a thrombotic event is not diagnosed, it causes suboptimal clinical symptoms management often. This aspect is very evident when an ischemic event involves part of the intestine as it can cause not specific symptoms such as diarrhea or chronic colitis whose ischemic nature does not recognize; at the same time, major intestinal ischemic events can be fatal. Moreover, the diagnosis of ischemic origin is even more complicated if bowel microvessels are involved, which give edema or thickening as the only detectable sign. Many commonly used anticancer agents are responsible for increased ischemic risk, including cisplatin, gemcitabine, and bevacizumab, which are also used in combination with many malignancies treatment. All these medications could cause intestinal damage on unknown ischemic epithelial injury. 5-fluorouracil and irinotecan-induced diarrhea or docetaxel and anti-PD-L1-induced colitis represent two combinations of drugs that might lead to intestinal damage that are much more frequent than highlighted in the literature.

Many different pathological mechanisms can cause bowel disease or ischemia; CT is the gold standard to diagnose pathological bowel features in the emergency setting because it allows detection of vascular anatomy and secondary signs of mesenteric ischemia, with high sensitivity and specificity (82–96% and 94%, respectively) [74–80]. It is established that oncologic patients are almost exclusively examined by CT to detect their “tumor burden.” Hence, it is possible to discover intestinal pathological features in asymptomatic patients during the planned follow-up.

We believe that CT is the primary imaging technique with a high temporal resolution that can provide a specific diagnosis of chemotherapy-induced bowel ischemia. CT could reveal also a fearsome complication that might occur in the bowel ischemia pathological process represented by bowel perforation. A hole through the wall of the intestines can develop; this results in the contents of the intestine leaking into the abdominal cavity, causing peritonitis. An interesting study conducted by Bagdwell and his group examined the management of perforation and the associated outcomes in patients with bevacizumab-associated bowel perforation. A bevacizumab-associated perforation on CT [6]. Bowel perforation was detected on CT in 24 patients of a total of 1442 patients showing that it represents however a rare complication; 23 of 24 were also treated with chemotherapy regimens. 83% of patients had only abdominal pain and many others were asymptomatic; in these case they were able to avoid surgery especially because the rapid and focused detection of bowel perforation on CT examination [6]. Risk factors for bevacizumab-related perforation could be represented by endoscopy executed 30 days before beginning treatment, previous adjuvant radiotherapy, long-term nonsteroidal anti-inflammatory drug (NSAID) therapy, peptic ulcer disease, diverticulosis, and previous surgery. The primary limits of this study, according to the authors, is represented by the lack of registries that may help to assess the true incidence of bowel perforation in these diseases [81–84].

Nivolumab determined small bowel obstruction and perforation in a patient that was on long-term therapy for metastatic non-small cell lung cancer that was confirmed after surgery [62]. This particular case report shows that anti-PD-1 antibody-related bowel inflammation may induce also stricture and bowel obstruction.

Therefore, it seems to be very important for radiologists to be aware as much as possible of the various chemotherapy treatment and pathological mechanisms that can promote bowel ischemia and perforation in the oncologic setting. Radiologists should talk to oncologists in multidisciplinary teams to manage together the clinical-therapeutic pathway of these patients; according to us, the knowledge of medical history in the oncologic setting is mandatory because, for example, patients could also be subjected to radiation before our observation so in this case the intestinal illness maybe not related to the therapeutic regimen. Patients with risk factors that could develop bowel perforation (history of diverticulitis, peptic ulcer disease, prior radiation exposure, previous bowel surgery) should be detected before therapy [85–87]. The radiologist should diagnose drug related-bowel toxicity because imaging manifestation of the drug toxicity may often take place before a patient develops symptoms [88, 89]. Rapid detection is essential because pneumatosis and perforation could be often treated conservatively with the interruption of therapy and supportive care. Thanks to CT reproducibility, as shown in our clinical case, radiologists can also examine patients after the suspension of the drug to evaluate if medical treatment has been definitely curative or not [90, 91].

## Conclusions

Interpretation of imaging in oncologic patients has become progressively more complicated in the context of “target therapy” and thanks to the increasing number and types of therapies provided. Radiologists should know this variety of antiangiogenic treatments and immunotherapy regimens first because they can determine atypical features of tumor response and then also because of their eventual bowel toxicity. To avoid emergency surgery and significant risk of mortality among chemotherapy-induced bowel ischemia, it is necessary to give prompt diagnoses detecting drug toxicities; the dialogue with the clinician could be helpful for radiologists for this issue. After all the recognition of risk factors for chemotherapy-associated bowel ischemia and perforation will depend on the assessment of large clinical trials and observational studies.
